# Supplementary material for: Patellar Tendinopathy—Does Injection Therapy Have a Role? A Systematic Review of Randomised Control Trials
Source: J Clin Med. 2022 Apr 3;11(7):2006. doi: 10.3390/jcm11072006 (PMC8999520; doi:10.3390/jcm11072006)
Supplement: Supplementary file 1 [file jcm-11-02006-s001.zip › Supplementary Table S1.pdf]

Supplementary Table S1. PICOS criteria used to define the research question

Acronym	Definiton	Description
**P**	Participants	Individuals diagnosed with patellar tendinopathy
**I**	Intervention	Administration of injections
**C**	Comparison	The comparison can occur through a placebo group or a group<br>that has received a treatment other than injection
**O**	Outcomes	Either patient-reported outcomes or biological and clinical markers of the tendon healing
**S**	Study design	Randomized Clinical Trials
