# Supplementary material for: Patellar Tendinopathy—Does Injection Therapy Have a Role? A Systematic Review of Randomised Control Trials
Source: J Clin Med. 2022 Apr 3;11(7):2006. doi: 10.3390/jcm11072006 (PMC8999520; doi:10.3390/jcm11072006)
Supplement: Supplementary file 1 [file jcm-11-02006-s001.zip › Supplementary Table S2.pdf]

Supplementary Table S2: Database formulas during the literature search

---

PubMed Search Formula

(Patella) OR (patellar)) OR (patellar tendon)) AND (Tendinopathy)) OR (tendinitis)) OR (tendinosis)) OR (patellar tendinopathy)) OR (jumpers knee))) AND (Injection)) OR (platelet-rich plasma)) OR (platelet rich plasma)) OR (corticosteroid)) OR (autologous blood)) OR (sclerosing)) OR (dry needling)) OR (hyaluronic acid)) OR (aprotinin)) OR (high volume injection)) OR (prolotherapy)

WOS Search Formula

(ALL=(Patella) OR ALL=(patellar) OR ALL=(patellar tendon) AND ALL=(Tendinopathy) OR ALL=(tendinitis) OR ALL=(tendinosis)) OR ALL=(patellar tendinopathy)) OR ALL=(jumpers knee)) OR ALL=(Injection)) OR ALL=(platelet-rich plasma)) OR ALL=(platelet rich plasma)) OR ALL=(corticosteroid)) OR ALL=(autologous blood)) OR ALL=(sclerosing)) OR ALL=(dry needling)) OR ALL=(hyaluronic acid)) OR ALL=(aprotinin)) OR ALL=(high volume injection)) OR ALL=(prolotherapy)

SCOPUS Search Formula

(TITLE-ABS-KEY (patella) OR TITLE-ABS-KEY (patellar) OR TITLE-ABS-KEY (patellar AND tendon) AND TITLE-ABS-KEY (tendinopathy) OR TITLE-ABS-KEY (tendinitis) OR TITLE-ABS-KEY (tendinosis) OR TITLE-ABS-KEY (patellar AND tendinopathy) OR TITLE-ABS-KEY (jumpers AND knee) AND TITLE-ABS-KEY (injection) OR TITLE-ABS-KEY (platelet-rich AND plasma) OR TITLE-ABS-KEY (platelet AND rich AND plasma) OR TITLE-ABS-KEY (corticosteroid) OR TITLE-ABS-KEY (autologous AND blood) OR TITLE-ABS-KEY (sclerosing) OR TITLE-ABS-KEY (dry AND needling) OR TITLE-ABS-KEY (hyaluronic AND acid) OR TITLE-ABS-KEY (aprotinin) OR TITLE-ABS-KEY (high AND volume AND injection) OR TITLE-ABS-KEY (prolotherapy))
